# Network Pharmacology and In Vivo Validation Reveal Therapeutic Effects of Sutaehwan in Polycystic Ovary Syndrome by Modulating AMH-AMHR2 Signaling Pathway

**DOI:** 10.1007/s43032-025-02027-x

**Published:** 2025-12-13

**Authors:** La Yoon Choi, Sujin Kwon, Sunju So, Yong-Deok Jeon, Dae Yong Kim, Mi Hye Kim

**Affiliations:** 1 College of Korean Medicine, Woosuk University, Jeonju, 54986 Republic of Korea; 2 Department of Korean Pharmacy, Woosuk University, Wanju, 55338 Republic of Korea

**Keywords:** Polycystic ovary syndrome, Sutaehwan, Korean medicine, Anti-Müllerian hormone

## Abstract

This study aimed to investigate the therapeutic effects and underlying mechanisms of Sutaehwan (STH), a traditional herbal formula, in polycystic ovary syndrome (PCOS), with a focus on anti-Müllerian hormone (AMH)-driven ovarian dysfunction. A network pharmacology approach was used to predict STH-related molecular targets and their intersection with PCOS-associated genes. GO and KEGG enrichment analyses were conducted on 45 overlapping genes. Then, in vivo validation was performed in a letrozole-induced PCOS rat model treated with various doses of STH (0.45, 0.9, 1.8 mg/kg) or metformin (500 mg/kg). Key endpoints included estrous cycle, ovarian morphology, serum hormone levels, and expression of AMH/AMHR2. Network analysis revealed significant enrichment in pathways related to ovarian steroidogenesis and cell cycle regulation. Daily administration of letrozole induced classical PCOS phenotypes including increased cystic follicles, elevated AMH/testosterone levels, and disrupted estrous cycles. STH treatment dose-dependently restored ovulatory function, reduced cystic structures, and normalized corpus luteum volume and endometrial thickness. STH also significantly downregulated AMH and AMHR2 expression at both of transcriptional and protein levels, particularly at the 1.8 mg/kg dose, with effects comparable to those of metformin. STH exerts therapeutic effects in a PCOS model by targeting AMH-mediated ovarian dysfunction. Through combined systems pharmacology and experimental validation, this study supports the potential of STH as a multi-target, endocrine-modulating therapy for PCOS, particularly in cases characterized by elevated AMH activity.

## Introduction

Polycystic ovary syndrome (PCOS) is one of the most common endocrine disorders, affecting approximately 5 ~ 10% of women of reproductive age [1]. It is diagnosed when two or more of the following features are present including hyperandrogenemia, chronic oligoovulation or anovulation and polycystic ovaries [2]. The incidence of PCOS has been rising in recent years, driven by a combination of genetic predisposition and environmental factors such as obesity, dietary habits, and metabolic disorders [3]. Especially, Metabolic abnormalities associated with PCOS have been shown to begin even before puberty [4]. In addition, PCOS is recognized as a systemic condition associated with metabolic and cardiovascular diseases [5], not just a reproductive disease, and is being redefined as a disease that requires long-term health care.

In recent years, several studies have been conducted to elucidate the pathophysiology and underlying mechanisms of PCOS. However, its etiology remains unclear despite multiple hypotheses, including insulin resistance, dysregulation of androgen production in the adrenal or ovary, and impaired secretion of gonadotropin-releasing hormone [6]. Among the proposed mechanisms, hyperinsulinemia and hyperandrogenemia are particularly significant, as they disrupt normal follicular development and contribute to metabolic complications such as type 2 diabetes and endometrial cancer in a substantial proportion of women with PCOS [7]. Lately, elevated levels of anti-Müllerian hormone (AMH), which are frequently observed in women with PCOS, reflect impaired folliculogenesis and have emerged as a potential biomarker for the diagnosis and assessment of the syndrome [8].

To manage PCOS-related metabolic disturbances, both weight reduction through lifestyle intervention is primarily recommended for its cost-effective and non-surgical option [9]. Change of body weight and body mass index (BMI) by lifestyle modification in PCOS women is reported to improve the free androgen index and glucose tolerance. Standard pharmacological interventions for PCOS typically include ovulation inducers (e.g., clomiphene citrate, letrozole), insulin sensitizers (e.g., metformin), and combined oral contraceptives [10, 11]. Metformin, a biguanide commonly prescribed to improve insulin sensitivity, also helps regulate androgen levels and restore ovulatory function [11]. Despite their widespread use, these treatments largely aim to alleviate symptoms rather than correct the underlying endocrine and metabolic dysfunctions [10, 12]. They are also commonly associated with side effects such as gastrointestinal disturbances, vitamin B12 deficiency, and systemic complaints including fatigue, headache, and joint pain. Furthermore, none of the currently available pharmacological options has proven effective as a definitive treatment for PCOS [13, 14]. The lack of a curative approach in existing treatments underscores the importance of developing therapies that directly target the fundamental mechanisms of PCOS.

Classical books of Traditional Korean Medicine, such as *Donguibogam*, *Bonchogangmok* and *Bangyakhappyeon*, describe Sutaehwan (STH) as a representative prescription used to stabilize the fetus and prevent miscarriage [15–17]. It is composed of herbal ingredients including *Cuscuta chinensis*, *Taxillus chinensis*, *Phlomis umbrosa* and *Asini corii colla* [16]. These herbs are traditionally used to tonify the kidney and nourish qi and blood, thereby supporting the maintenance of pregnancy [17]. Recently, clinical studies involving STH have reported its potential in improving endometrial stability, enhancing uterine blood flow, and exerting antioxidant effects, contributing to increased pregnancy maintenance rates and reduced miscarriage risk [17]. Additionally, mechanisms related to implantation stability, immune modulation, and uterine perfusion improvement have been proposed [17]. Although it is hypothesized that STH may have therapeutic potential for PCOS based on these findings, definitive preclinical evidence is still lacking.

Despite the long history of clinical application and reported effectiveness of traditional herbal medicines, their mechanisms of action and therapeutic efficacy remain insufficiently validated within the framework of modern biomedical science. Given that these medicines often exert effects through multi-compound, multi-target, and multi-pathway interactions, this study employs a network pharmacology approach to predict the potential mechanisms by which the components of STH may act in the treatment of PCOS. To further investigate its therapeutic potential, an animal model of PCOS will be employed to assess the therapeutic effects of STH.

## Materials and Methods

### Constitute Compound and Disease-Associated Gene Set

The herbal ingredients of STH were confirmed using the Korean Pharmaceutical Information Center (https://www.health.kr/main.asp). All compounds in STH were collected from public databases TM-MC (https://tm-mc.kr/). Genes associated with each compound were retrieved from CTD (https://ctdbase.org/). Also, PCOS-related genes were obtained from DisGeNET (https://www.disgenet.org). Overlapped genes between the herb-derived genes and disease-associated genes were selected. Protein–protein interactions were analyzed using the STRING database (https://string-db.org/) for the genes that overlapped between STH compound targets and PCOS-associated genes, with the confidence score threshold set to 0.7.

## Gene Ontology and Kyoto Encyclopedia of Genes and Genomes Enrichment Analysis

Gene ontology (GO) including Biological Process (BP), Molecular function (MF), and Cellular Component, and Kyoto Encyclopedia of Genes and Genomes (KEGG) enrichment analyses were performed using ShinyGO v0.82 (https://bioinformatics.sdstate.edu/go/) [18] to identify the biological functions and regulatory pathways associated with the 45 target genes. Statistically significant categories were selected using a cutoff of *p* < 0.001. Enrichment results were visualized using SRPLOT (https://www.bioinformatics.com.cn/en).

## Sample Preparation

*Cuscuta chinensis* 120 g, *Taxillus chinensis* 60 g, *Phlomis umbrosa* 60 g and *Asini corii colla* 60 g were extracted with distilled water at 100℃ for 2 h. All herbs have undergone quality standard testing in compliance with hGMP guidelines. The yield of the extract after freeze-drying was 16.34%. A preliminary pilot study in rats indicated that 1.8 mg/kg of STH extract yielded optimal therapeutic effects in improving ovarian morphology and hormone levels without observable toxicity. Based on these findings, the highest daily dose was set at 1.8 mg/kg. Accordingly, the extract was dissolved in distilled water at concentrations of 0.45, 0.9, and 1.8 mg/kg and administered to the rats.

## Animal Experiments

Female Sprague Dawley rats (80 ~ 100 g) were purchased from Damul Science (Daejeon, Republic of Korea). The animals were housed in an animal facility with a controlled environment (12-hour light/dark cycle, temperature 22 ± 2 °C, and humidity 55 ± 5%) and allowed free access to standard chow and water. They were acclimated for one week prior to the experiment. All animal procedures were approved by the Institutional Animal Care and Use Committee (IACUC) of Woosuk University (Approval No.: WS-2024-22). Prior to the experiments, an a priori power analysis using G Power 3.1.9.7 indicated that a minimum of 10 animals per group would be sufficient to achieve a statistical power of 0.80 at an effect size of 0.5 and α = 0.05. The rats were randomly divided into six groups (*n* = 10); CON, normal control; LV, letrozole-induced PCOS model; Met, the PCOS model group treated with metformin; S0.45, the PCOS model group treated with STH 0.45 mg/kg; S0.9, the PCOS model group treated with STH 0.9 mg/kg; the PCOS model group treated with S1.8, STH 1.8 mg/kg. PCOS was induced by daily oral administration of 1 mg/kg letrozole (MedChemExpress, NJ, USA) dissolved in 1% carboxymethylcellulose (CMC, 1 mL) for 4 weeks. The PCOS control group (LV) received the same letrozole induction followed by vehicle (saline) administration without any therapeutic agents. The positive control group received metformin (Cayman Chemical, Ann Arbor, MI, USA) at a dose of 500 mg/kg daily for 4 weeks. STH was administered daily for the same duration. Estrous cycles were monitored via vaginal smears for 3 days prior to sacrifice. After 4 weeks of treatment, the animals were euthanized for further analysis. To further verify adequacy, a post hoc power analysis was conducted based on the actual sample size (*n* = 10 per group, total *n* = 60), which yielded a power of 0.825, confirming that the chosen group size provided sufficient statistical sensitivity to detect meaningful differences across groups.

## Histological Analysis

Ovarian and uterine tissues were harvested and fixed in 10% formaldehyde for 24 h. Subsequently, dehydrated using graded series of ethanol and xylene. After embedding in paraffin, the specimens were sectioned 4 μm with a microtome. Hematoxylin and eosin (H&E) staining was performed to examine cystic follicle in the ovary, and the morphology of endometrial glands and endometrium thickness in uterus. Images were acquired using a Leica Application Suite (LAS) Microscope Software (Leica Microsystems Inc., Wetziar, Land Hessen, Germany) with a digital camera at 40× and 100× magnification. For quantitative evaluation, follicles were counted in at least five randomly selected fields per ovary. Image analysis was performed using ImageJ software (NIH, Bethesda, MD, USA).

### Immunofluorescence Assay

The tissue sections were subjected to standard deparaffinization and rehydration steps, followed by heat-induced antigen retrieval in citrate buffer (pH 6.0). Endogenous peroxidase activity was blocked by incubating the sections with 3% hydrogen peroxide. To minimize nonspecific binding, sections were blocked with normal horse serum. Slides were then incubated overnight at 4℃ in the dark with an anti-AMH primary antibody 1:400 dilution (Santa Cruz Biotechnology, TX, USA). After washing, the sections were incubated with a TRITC-conjugated goat anti-mouse IgG secondary antibody (Thermo Fisher Scientific, MA, USA) at room temperature for 1 h under light-protected conditions. Nuclei were counterstained using DAPI-containing antifade mounting medium (Vector Laboratories, Inc., CA, USA). Stained sections were examined using a ZEN-blue edition software (ZEN 2.6., Carl Zeiss Microscopy GmbH, Thornwood, NY) to assess the localization and expression of AMH in the ovarian tissue. Images were captured at 400× magnification to provide clear visualization of follicular structures. For quantitative analysis, ImageJ software (NIH, USA) was used to measure the mean fluorescence intensity (MFI) of AMH signals, which was normalized to background intensity. At least five randomly selected fields per section were analyzed for each sample to ensure reproducibility.

## Protein Immunoblot Analysis

Total protein was extracted from ovary tissues using radioimmunoprecipitation assay (RIPA) buffer supplemented with protease inhibitor cocktails. Protein concentrations were measured using the Bradford assay. Lysates were denatured at 98 °C for 5 min, and 10 µg of protein per sample was loaded onto a 10% SDS-polyacrylamide gel. After electrophoresis, proteins were transferred to polyvinylidene fluoride (PVDF) membranes and blocked with 3% bovine serum albumin (BSA) at room temperature for 1 h. Membranes were then incubated overnight at 4 °C with the following primary antibodies (1:1000 dilution in TBS-T): AMH (SC-166752, Santa Cruz Biotechnology, TX, USA), AMHR2 (AF1618, R&D systems, MN, USA). After washing, membranes were incubated with HRP-conjugated secondary antibodies for 1 h at room temperature. Bands were visualized using enhanced chemiluminescence (ECL) reagents and detected with a chemiluminescence imaging system (Cytiva, Incheon, Korea). Band intensities were quantified using ImageJ software (NIH, Bethesda, MD, USA).

## Quantitative Polymerase Chain Reaction (qPCR)

Ovarian tissues collected from rats were processed for RNA isolation. Approximately 30 mg of frozen tissue was lysed in 1 mL of Easy BLUE™ reagent (iNtRON Biotechnology,

Seongnam, Republic of Korea) following the manufacturer’s instruction. The RNA pellet was dissolved in 100 µL of diethyl pyrocarbonate-treated water, and its purity was spectrophotometrically assessed (A260/280 ≥ 1.9). Subsequently, 1 µg of total RNA was used with the PrimeScript™ RT Reagent Kit (TaKaRa Bio Inc., Kusatsu, Japan) according to the manufacturer’s instructions. qPCR was performed using TB Green^®^ Premix Ex Taq™ II (TaKaRa Bio Inc., Kusatsu, Japan) on a Thermal Cycler Dice^®^ Real-Time PCR System (TaKaRa Bio Inc., Kusatsu, Japan)(forward and reverse; sequences listed in Table 1). All steps were proceeded following the manufacturer’s protocol. Melting curve analysis was conducted after amplification to verify the specificity of the PCR products.

**Table 1:** Real-time PCR primer sequences

Gene name	Sequence	
	Forward (5’ to 3’)	Reverse (5’ to 3’)
*AMH*	CTTTCTGTTTGGCTCTGATTCCCG	GTGGGTGGCAGCAGCACTAGG
*AMHR2*	CAACATCCCTTCCTCTTGGAG	CGTCCCAGCAATCTTCCA
*ACTB*	TGTCCACCTTCCAGCAGATGT	AGCTCAGTAACAGTCCGCCTAGA

### Statistical Analysis

Statistical analysis was performed using GraphPad Prism version 8.0.2 (Boston, MA). Data were analyzed by one-way analysis of variance (ANOVA), followed by Tukey’s post hoc test for multiple comparisons. Results are presented from three independent, and differences were considered statistically significant at *p* < 0.05.

## Results

### Identification of Common Targets between STH and PCOS

**Fig. 1:**
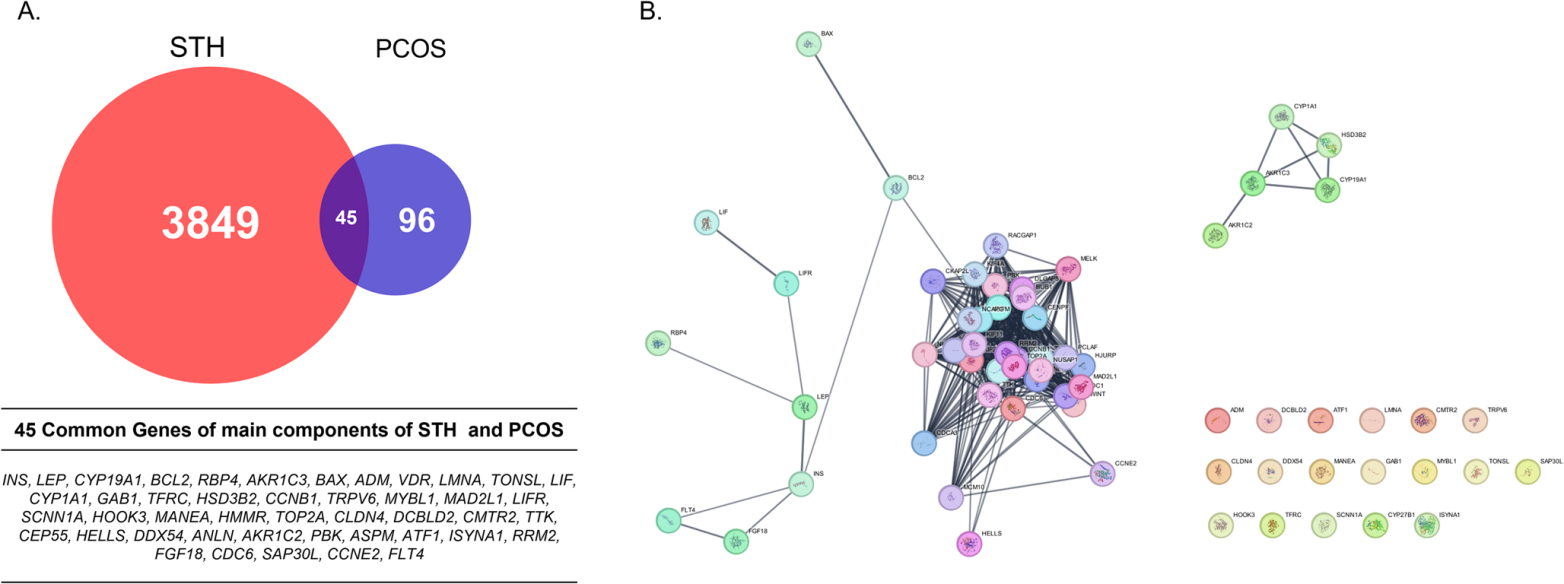
Venn Diagram and Network Analysis of Gene Interactions (**A**) Venn diagram showing 3,894 genes of STH and 141 genes of PCOS, with 45 overlapping genes. (**B**) Network interaction of 45 overlapping genes, displaying 65 nodes and 436 edges

A total of 3,894 genes associated with STH compounds and 141 genes related to PCOS were compiled from public databases. Among these, 45 overlapping genes were identified through Venn diagram analysis (Fig. 1A). To explore the functional associations among these overlapping genes, a protein–protein interaction (PPI) network was constructed using the STRING database with a confidence score cutoff of 0.7. The resulting network contained 65 nodes and 436 edges, suggesting strong interconnectivity among the targets (Fig. 1B).

### Functional Enrichment of Overlapping Target Genes

**Fig. 2:**
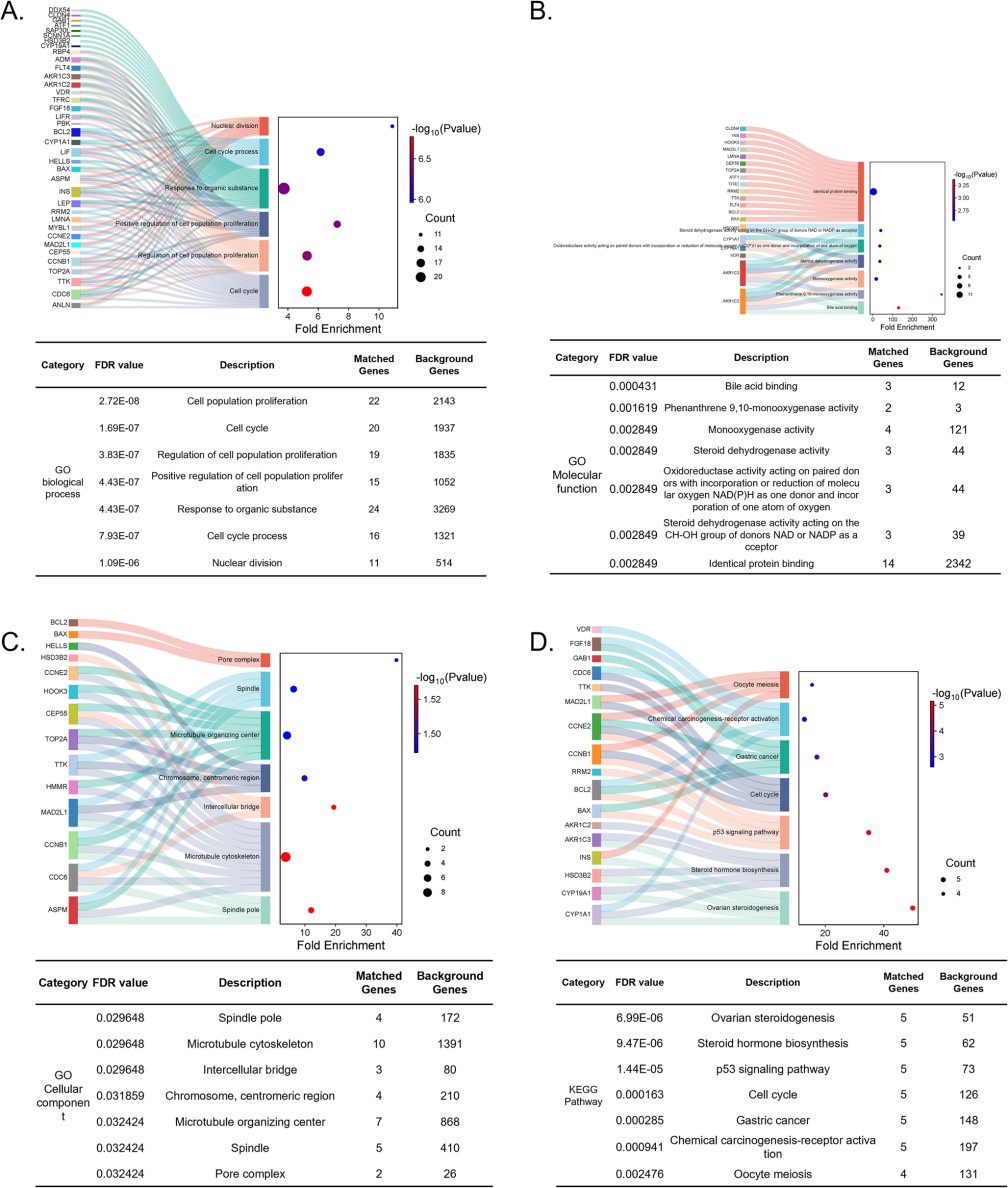
Functional Enrichment Analysis of Gene Sets. (**A**) GO biological process analysis showing functional enrichment in processes such as nuclear division and cell cycle regulation. (**B**) GO molecular function analysis highlighting key molecular functions, including binding and enzyme activity. (**C**) Cellular component analysis identifying gene enrichment in specific cellular locations such as the nucleus and cytoplasm. (**D**) KEGG pathway enrichment analysis displaying significant pathways, including ovarian mesoderm and hormone biosynthesis

In Fig. 2, GO and KEGG enrichment analyses were conducted to explore the biological functions of the 45 overlapping genes. GO analysis revealed significant enrichment in processes such as nuclear division, mitotic cell cycle, and protein kinase activity. KEGG analysis indicated that the targets were involved in several PCOS-related pathways, including ovarian steroidogenesis, ovarian mesodermal development, and endocrine regulation.

### Effects of STH on Estrous Cycle Recovery

**Fig. 3:**
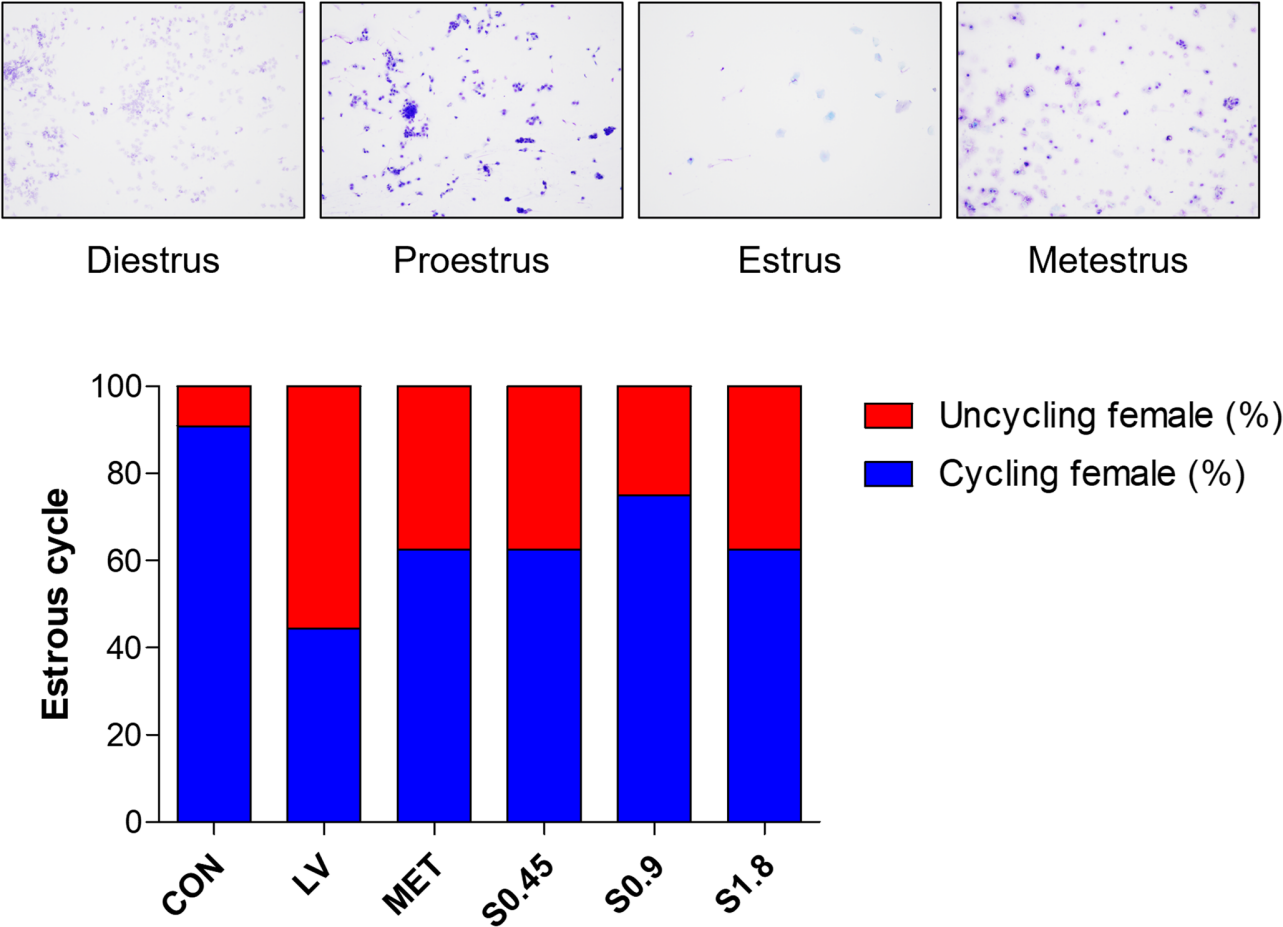
Estrous cycle assessment by vaginal smear. Vaginal smears were performed to monitor the estrous cycle in the experimental groups.The estrous stages were observed across the control (CON), LV-induced PCOS (LV), andsample treatment groups. The cycle stage was determined based on the presence ofcornified epithelial cells (indicative of estrus) or nucleated cells (proestrus)

Vaginal smear analysis showed that rats in the LV-induced group exhibited prolonged diestrus and irregular estrous cycles, indicating ovulatory dysfunction. In contrast, STH-treated groups displayed partial recovery of estrous cyclicity, with increased frequency of proestrus and estrus stages. These findings indicate that STH treatment may help restore reproductive cycle regularity in PCOS-induced rats (Fig. 3).

### Improvement of Ovarian Morphology and Weight

**Fig. 4:**
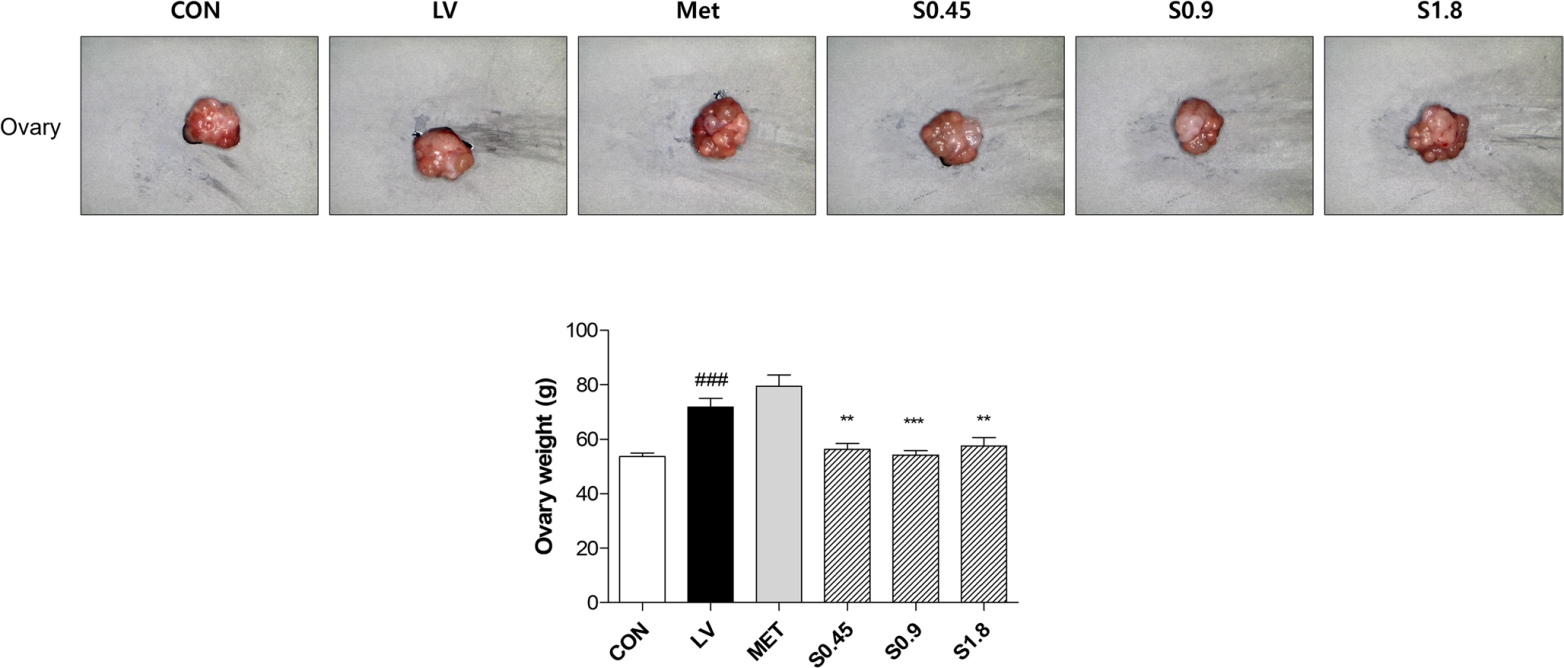
Ovary morphology and weight. Representative images of ovary morphology and corresponding weight measurements are shown for the CON, LV-induced PCOS, and sample treatment groups. Ovary weights were significantly increased in the LV group compared to CON, and the sample treatment groups showed a notable reduction in weight. Data are presented as the mean ± SD. Statistical significance is indicated by p values as ^**^ *p* < 0.01, ^***^p < 0.001

LV-induced PCOS rats exhibited enlarged, pale ovaries with indistinct follicular morphology and a 1.3-fold increase in ovarian weight compared to the control group. Following administration of STH at all tested doses, ovarian weight was significantly reduced, approaching 0.8-fold relative to the LV group (Fig. 4).

### Histological Improvement in Ovary and Uterus

**Fig. 5:**
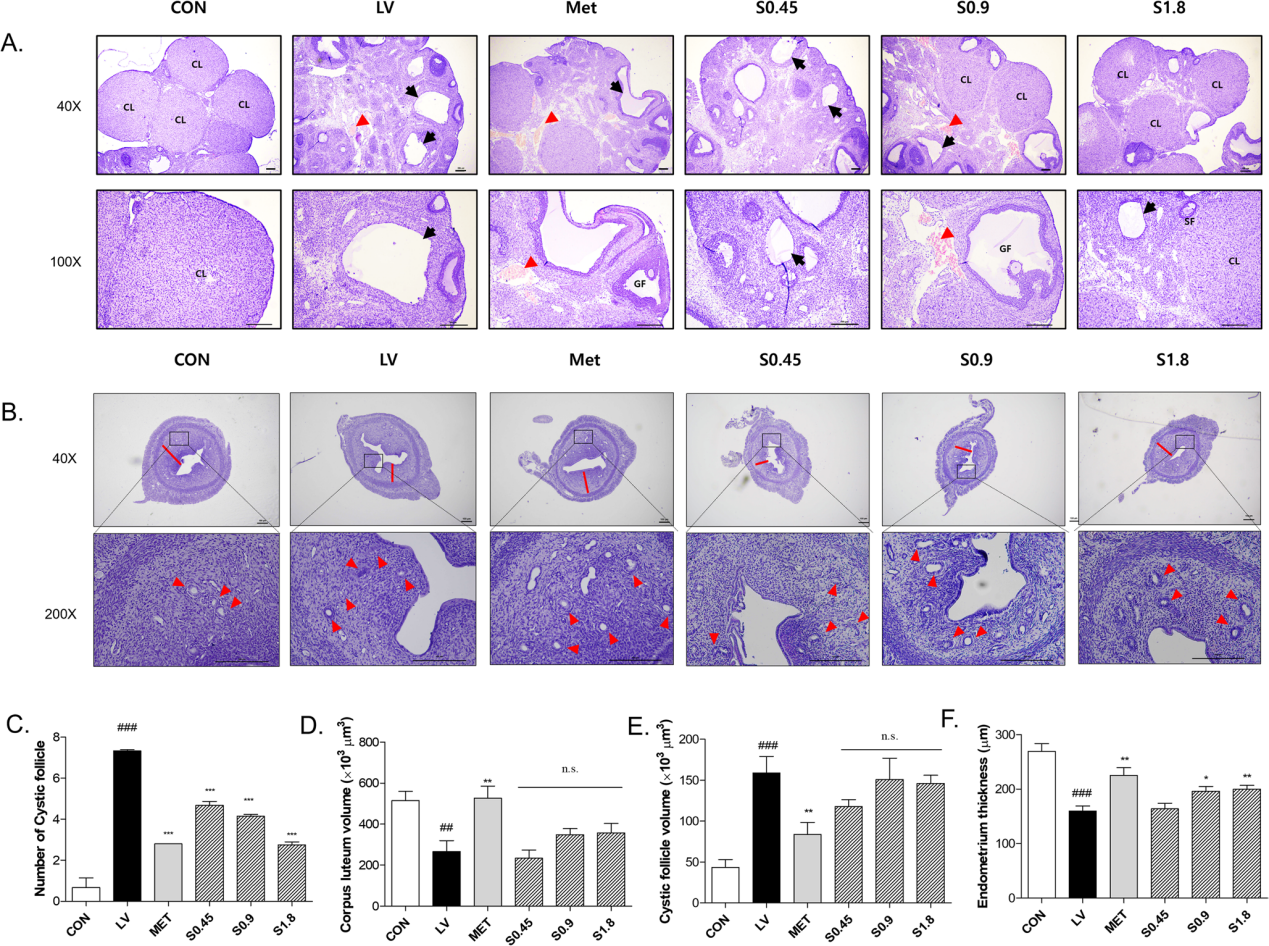
Histological evaluation of ovarian and uterine tissues in the LV-induced PCOS model. (**A**) Representative H&E-stained ovarian Sect. (40× and 100×) show morphological changes across groups, including cystic follicles and corpus luteum development. (**B**) H&E-stained uterine Sect. (40× and 200×) illustrate changes in endometrial structure and glandular morphology. (**C**) Quantification of cystic follicle numbers. (**D**) Corpus luteum volume. (**E**) Cystic follicle volume. (**F**) Endometrial thickness across groups. Data are presented as mean ± SD. # indicates comparison between the CON and LV groups, while * denotes comparison between the LV and treatment groups(^*^ *p* < 0.05, ^**^ *p* < 0.01, ^***^p < 0.001)

In the LV group, the average number of cystic follicles increased by approximately 11-fold compared to the CON group. MET group showed a reduction to 0.38-fold compared to LV. Also, STH treatment reduced this value to 0.64, 0.57, 0.38-fold of S0.45, S0.9, S1.8, respectively, relative to the LV group (Fig. 5A, C).

Corpus luteum volume was significantly diminished in the LV group, falling to 52% of the control value. Treatment with STH restored the volume, reaching 1.30, 1.34, and 1.34 times that of LV in the respective doses of STH groups. In comparison, the MET group showed the most prominent increase, with values rising to nearly 1.97 times that of LV (Fig. 5A, D).

In Fig. 5A and E, compared to the CON group, the LV group demonstrated a substantial enlargement in cystic follicle volume, showing an increase of approximately 3.66-fold. Administration of MET treatment also suppressed this enlargement, with cystic follicle volume reduced to 0.53 times the level observed in LV rats. Likewise, STH led to a dose-dependent decline, reducing the volume to 0.74, 0.95, and 0.92 times relative to LV.

Endometrial thickness was markedly reduced in the LV group, showing only 59% of the control level. This reduction was alleviated by STH treatment, which restored endometrial thickness to 1.10, 1.23, and 1.29 times that of the LV group in S0.45, S0.9, and S1.8, respectively. In addition, the MET group exhibited significant recovery, with thickness values reaching 1.41 times that of LV (Fig. 5B, F).

### Visualization of Ovarian AMH Expression

**Fig. 6:**
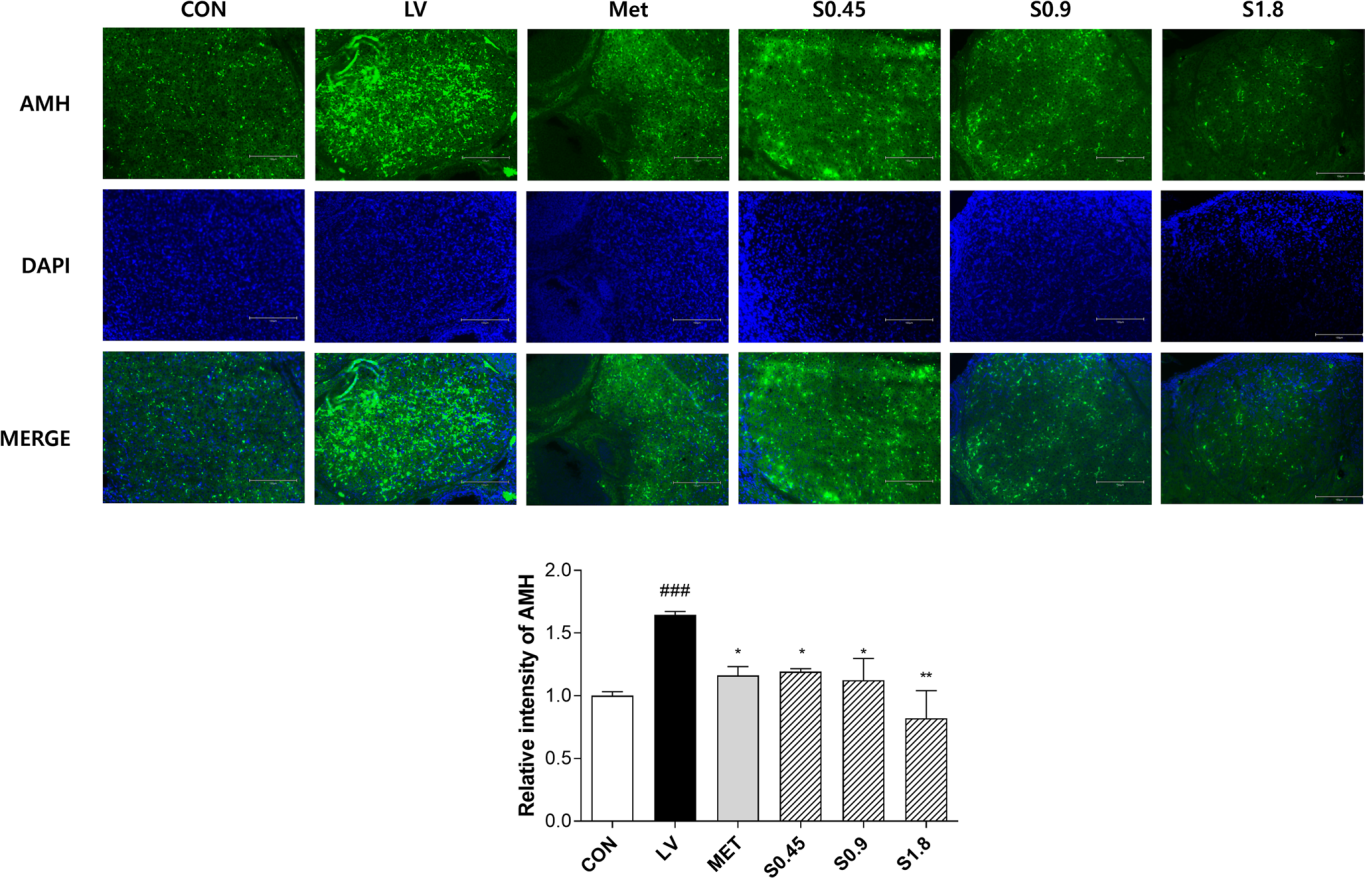
AMH expression in ovarian tissues via immunofluorescence staining. Representative immunofluorescence images showing AMH (green) expression in ovarian follicles from each experimental group. DAPI (blue) was used for nuclear counterstaining. Quantitative analysis of relative fluorescence intensity is presented. Data are presented as mean ± SD. # indicates comparison between the CON and LV groups, while * denotes comparison between the LV and treatment groups(^*^ *p* < 0.05, ^**^ *p* < 0.01, ^***^p < 0.001)

In Fig. 6, Immunofluorescence analysis was conducted to evaluate AMH protein expression in ovarian sections. The LV group exhibited a marked elevation in AMH signal intensity, reaching 1.65 relative to the control group. MET treatment moderately reduced this expression to 1.16. In contrast, STH administration led to dose-dependent suppression of AMH expression, with relative intensities of 1.19 (S0.45), 1.12 (S0.9), and 0.82 (S1.8).

### Suppression of AMH and AMHR2 Protein Expression by STH Treatment

**Fig. 7:**
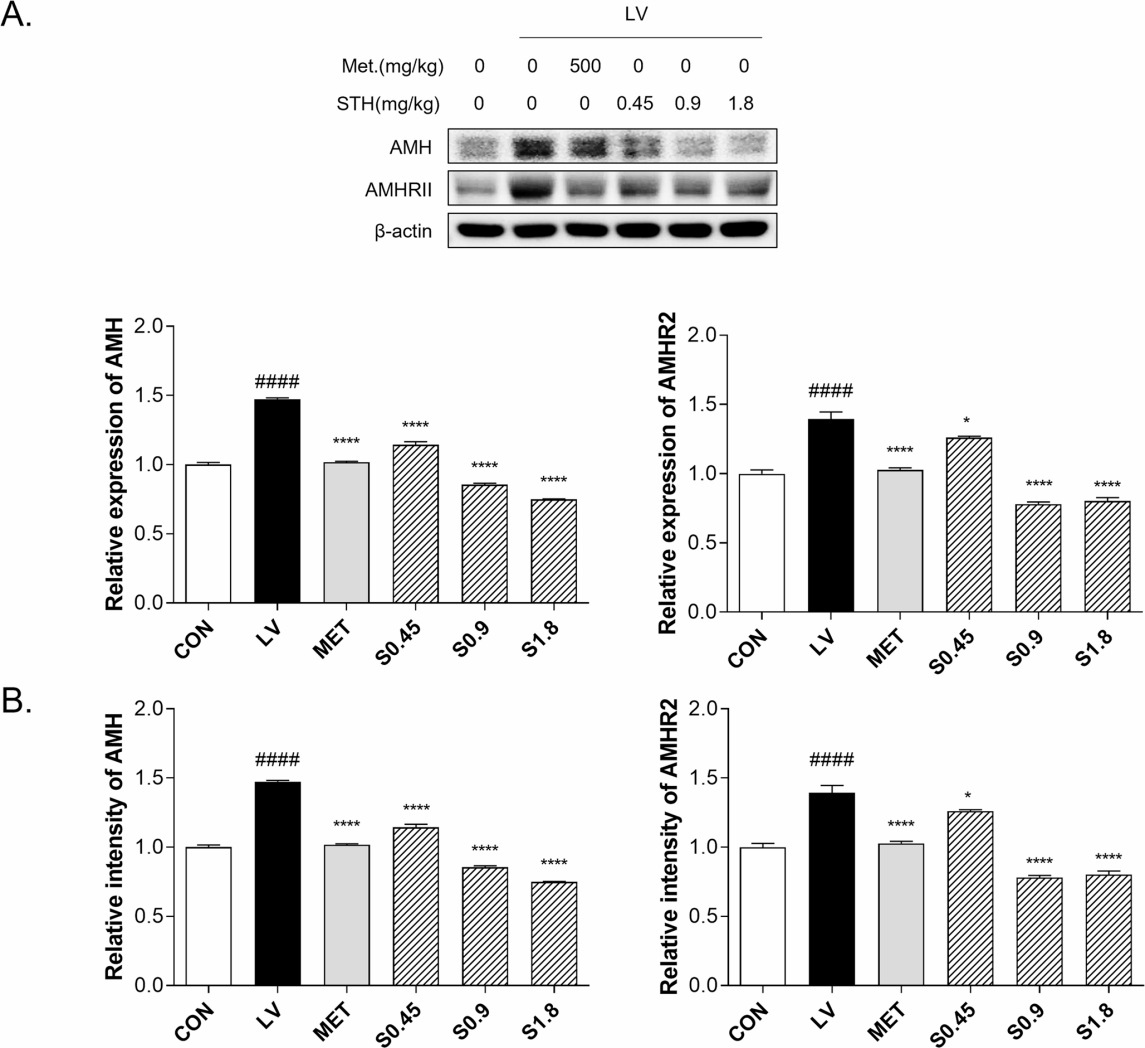
Effects of STH on protein and gene expression in LV-induced PCOS rats. (**A**) Protein anti-Müllerian hormone (AMH) and AMHR2 levels were measured using western blot. (**B**) mRNA expression of AMH and AMHR2 in ovarian tissue was analyzed by qPCR. Data are presented as mean ± SD. # indicates CON vs. LV, * denotes LV vs. treatment (^*^ *p* < 0.05, ^**^ *p* < 0.01, ^****^p < 0.0001)

Western blot analysis revealed that the expression level of AMH protein was significantly increased in the LV group, showing a 1.47-fold elevation compared to the control. Treatment with metformin reduced AMH expression to 1.00-fold, returning it to a level comparable with the control group. Notably, STH treatment resulted in a dose-dependent reduction of AMH protein, with levels decreasing to 1.14-fold (S0.45), 0.85-fold (S0.9), and 0.75-fold (S1.8) relative to the LV group. These findings suggest that STH effectively suppresses the upregulation of AMH induced by letrozole administration. Similarly, AMHR2 protein levels were markedly elevated in the LV group, reaching 1.39-fold compared to the control. Metformin-treated rats showed a reduction to 1.02-fold. Administration of STH led to a significant decrease in AMHR2 protein levels in a dose-responsive manner, with relative intensities of 1.26 (S0.45), 0.78 (S0.9), and 0.80 (S1.8) compared to the LV group (Fig. 7A).

### Regulation of Ovarian Transcriptional Activity by STH Treatment

Quantitative PCR analysis revealed that ovarian *AMH* expression in the LV group was elevated to 1.47 times that of the control group. STH treatment led to a marked reduction in expression, with values decreasing to 0.61, 0.58, and 0.51 folds of S0.45, S0.9, S1.8, respectively. In comparison, the MET group reduced levels similar to the control group by treating metformin. For *AMHR2*, expression was also increased in the LV group (1.39 times relative to control). This upregulation was suppressed by STH administration, resulting in values of 0.90, 0.56, and 0.58 in the S0.45, S0.9, and S1.8 groups, respectively. MET treatment moderately lowered expression to 0.73 compared to LV (Fig. 7B).

## Discussion

This study aimed to elucidate the therapeutic potential of STH, a traditional Korean herbal formula, in the treatment of PCOS using a combined network pharmacology and in vivo experimental approach. Our investigation focused on AMH-driven ovarian dysfunction, given the increasing evidence implicating elevated AMH levels as a hallmark and potential therapeutic target in PCOS. Through systematic compound–target–disease mapping, network analysis, and experimental validation using a letrozole-induced PCOS rat model, we demonstrated that STH exerts significant regulatory effects on AMH signaling and associated ovarian pathophysiology.

Firstly, network pharmacology analysis identified 3,894 compound-associated genes from STH and 141 PCOS-related genes, among which 45 overlapping targets were selected. GO and KEGG pathway enrichment analyses revealed that these targets are mainly involved in biological processes critical to reproductive endocrinology, such as steroid hormone biosynthesis, ovarian mesodermal development, and cell cycle regulation. Notably, AMH and its receptor AMHR2 emerged as key nodes within the PPI network, supporting their mechanistic relevance. This computational prediction laid the foundation for experimental validation targeting the AMH axis.

In this study, letrozole administration successfully induced classical features of PCOS, including anovulation, irregular estrous cycles, increased ovarian weight, and cystic follicle accumulation. These phenotypic alterations were accompanied by markedly elevated serum testosterone and AMH concentrations, as well as increased expression of AMH and AMHR2 mRNA in ovarian tissues. These findings are consistent with previous reports that hyperandrogenism and AMH dysregulation are central contributors to impaired folliculogenesis in PCOS [19, 20]. Several herbal formulations have been investigated in PCOS models, demonstrating improvements in both reproductive and metabolic outcomes. For example, licorice ethanol extract has been reported to improve estrous cyclicity, normalize serum testosterone, and ameliorate ovarian morphology in letrozole-induced PCOS rats, suggesting its role in restoring endocrine balance and follicular development [21]. Similarly, lepidium sativum extract was shown to alleviate reproductive and developmental toxicity in a letrozole and high-fat diet-induced PCOS rat model, with notable improvements in follicular growth, estrous cycle regulation, and oxidative stress markers [22]. While these herbal approaches primarily target androgen excess, follicular maturation, and metabolic dysregulation, our findings provide direct evidence that STH reduces AMH and AMHR2 expression at both transcriptional and protein levels, accompanied by morphological improvements in ovarian tissue such as restoration of corpus luteum and reduction of cystic follicles. This mechanistic difference highlights the potential of STH not only to complement existing herbal therapies but also to provide a novel therapeutic angle focusing on ovarian folliculogenesis in PCOS. STH treatment reversed letrozole-induced PCOS-like symptoms in rats in a dose-dependent manner. Vaginal smear analysis revealed partial restoration of the estrous cycle, particularly in the proestrus and estrus phases, suggesting improved ovulatory function. Histological examination of ovarian tissues demonstrated a substantial decrease in the number and volume of cystic follicles and a concurrent increase in corpus luteum formation, indicating reactivation of follicular maturation and ovulation. Importantly, these improvements were accompanied by a significant reduction in both circulating AMH levels and ovarian AMH and AMHR2 expression, particularly at the highest tested dose of 1.8 mg/kg.

In detail, treatment with STH demonstrated effects comparable to metformin in specific morphological parameters, such as restoration of corpus luteum volume and suppression of cystic follicular structures. This suggests that STH not only alleviates endocrine imbalances but may also contribute to structural remodeling of the ovary, potentially enhancing its therapeutic applicability beyond metabolic correction. In addition, endometrial thickness, often reduced in PCOS and associated with implantation failure, was significantly restored by STH, further supporting its pro-fertility potential.

In addition, the AMH signaling pathway was significantly attenuated by STH treatment. The role of AMH in PCOS has been increasingly highlighted in recent years. AMH is produced by granulosa cells of small growing follicles and plays a key role in inhibiting initial follicular recruitment and modulating FSH sensitivity [23]. In PCOS, excessive AMH production has been linked to persistent antral follicle dominance and ovulatory failure [24]. Our findings that STH effectively reduces *AMH* and *AMHR2* expression at both transcriptional levels and protein levels indicate that this formula may directly counteract AMH-driven follicular arrest, thereby reactivating normal folliculogenesis. Immunofluorescence staining further supported the molecular and histological findings by visualizing the spatial distribution of AMH expression within ovarian follicles. In the LV group, intense AMH fluorescence signals were observed throughout the granulosa cell layer, reflecting aberrant overexpression consistent with disrupted folliculogenesis. In our study, STH administration resulted in a clear reduction in AMH immunoreactivity across the ovarian cortex, particularly at higher doses. This localized reduction of AMH protein expression suggests that STH may directly influence intrafollicular signaling environments known to contribute to follicular arrest. High intraovarian AMH levels have been implicated in the inhibition of follicle maturation and the persistence of small follicles [25], a hallmark of PCOS pathology. By downregulating AMH at the tissue level, STH may alleviate this intrafollicular blockade and facilitate the transition toward ovulatory follicle development [26]. These findings are in line with the systems pharmacology predictions, where AMH and AMHR2 were identified as central network nodes linking STH compounds to PCOS-associated molecular pathways. Together, these results reinforce the hypothesis that STH ameliorates PCOS not only through systemic hormonal regulation but also via localized modulation of pathogenic signals in ovarian microstructures.

This is consistent with the systems-level hypothesis derived from network pharmacology that STH acts via multi-target, multi-pathway mechanisms. Indeed, herbs in STH such as *Cuscuta chinensis* and *Taxillus chinensis* have been previously reported to possess endocrine-modulating and antioxidant properties, which may synergistically contribute to follicular recovery [27, 28].

## Conclusion

This study provides novel insights into the mechanism by which STH alleviates ovarian dysfunction in PCOS. By modulating AMH/AMHR2 signaling and restoring normal follicular architecture, STH exhibits both endocrine and histological benefits in a preclinical PCOS model. These findings not only support the traditional use of STH for gynecological disorders but also highlight the utility of network pharmacology in uncovering multi-target herbal mechanisms. STH may represent a promising complementary therapeutic option for PCOS, particularly in patients with AMH-dominant ovarian dysfunction.

## Data Availability

The datasets used and/or analyzed during the current study are available from the corresponding author on reasonable request.
